# Seatbelt: A Double-Edged Sword

**DOI:** 10.1155/2012/326936

**Published:** 2012-02-28

**Authors:** P. Raychaudhuri, N. K. Cheung, C. Bendinelli, M. Puvaneswary, R. Ferch, Rajendra Kumar

**Affiliations:** ^1^Department of Surgery, John Hunter Hospital, Newcastle, NSW 2305, Australia; ^2^Department of Neurosurgery, John Hunter Hospital, Newcastle, NSW 2305, Australia; ^3^Department of Surgery/Trauma Service, John Hunter Hospital, Newcastle, NSW 2305, Australia; ^4^Department of Radiology/Trauma Service, John Hunter Hospital, Newcastle, NSW 2305, Australia; ^5^Department of Paediatric Surgery, John Hunter Children's Hospital, Newcastle, NSW 2305, Australia

## Abstract

Intra-abdominal vascular injury due to blunt trauma is unusual in children. Due to its rarity, detailed reports dealing with its management are scarce in paediatric literature. Diagnosis of these injuries is challenging, and a high degree of awareness is necessary for rapid identification and treatment of these injuries. We report the case of a child with seatbelt sign and mesenteric vein injury due to blunt trauma to the abdomen during a motor vehicle accident where the seatbelt was incorrectly placed. She also sustained cervical vertebral injury. The pattern of injuries in children in these situations may differ from that found in adults. While seatbelts have undoubtedly saved many lives, awareness about correct placement of these restraints is extremely necessary.

## 1. Introduction

Seatbelts have saved the lives of many children involved in motor vehicle accidents [1]. However, if wrongly placed, it can cause abdominal wall bruising (referred to as the seatbelt sign) and injuries to the lumbar spine or intra-abdominal organs. Seatbelt sign (SBS) is uncommon and is found in about 1.3% of all restrained children involved in motor vehicle accidents. Those with abdominal wall bruising have been found to be 232 times more likely to sustain serious intra-abdominal injury compared to those without a bruise and nine times more likely to sustain lumbar vertebral injuries [2]. However, the spectra of abdominal injuries in children with SBS seems to be wider than those described in adult victims. Abdominal vascular injuries in these circumstances are rare in children [3]. We report the case of a nine-year-old girl who was the restrained, left-sided, and rear-seat passenger in a high-speed, side-on collision with SBS, injuries to the cervical spine, and tear of the superior mesenteric vein (SMV).

## 2. Case Presentation

**Figure 1:**
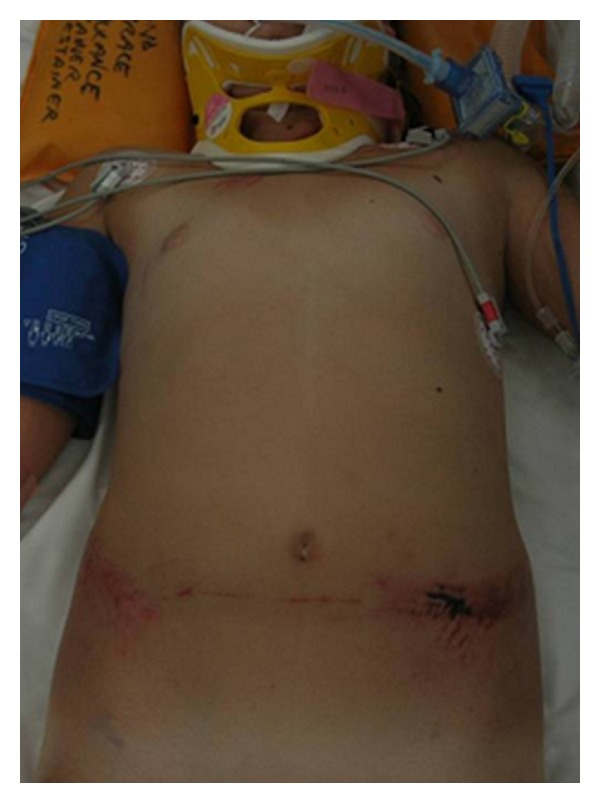
Seatbelt sign (note wrong position of the seatbelt).

**Figure 2:**
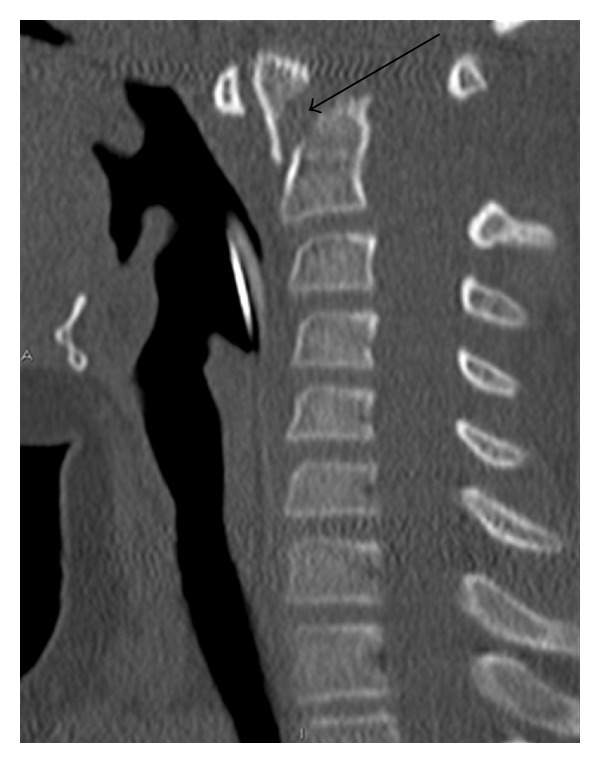
Type III displaced and angulated fracture of the odontoid process.

**Figure 3:**
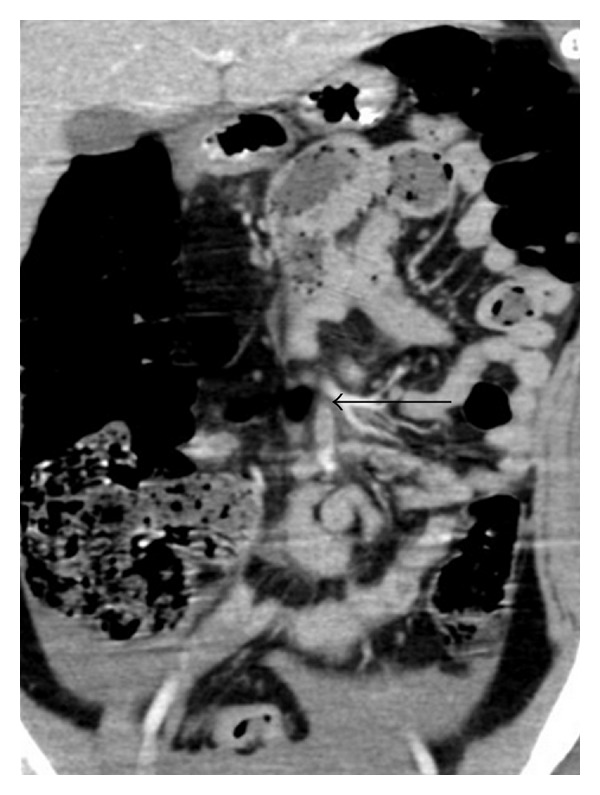
Coronal view arterial phase CT abdomen. Filling defect in main SMV (long arrow) and large amount of intraperitoneal blood.

The child was retrieved by helicopter from peripheral hospital to the Level 1 Trauma Center. On arrival in the emergency department, she was haemodynamically stable. Her abdomen was distended and tender with the presence of guarding. She also had marked seatbelt bruising on the anterior abdominal wall (Figure 1) and midline cervical spine tenderness. FAST scan demonstrated intra-abdominal free fluid. CT scan of the cervical spine revealed an anteriorly displaced transverse fracture of the odontoid process with subluxation of C2 on C3 vertebra (Figure 2). Abdominal CT scan confirmed the presence of free fluid in the abdomen and a filling defect in the superior mesenteric vein (Figure 3). Urgent trauma laparotomy with full cervical spine precautions was undertaken. Active bleeding from a tear of the SMV and one of its proximal branches was found. The tear of SMV was repaired with 5–0 prolene suture, and the torn branch was ligated. No other visceral injuries were identified on further exploration. The dens fracture was initially managed with a Miami J collar which was changed to halo traction on day 3. Open reduction with wiring was undertaken on the fourth day after injury as the halo traction was found to be inadequate. The patient progressed well following surgery and was discharged home 26 days after admission. At 12-month followup, she is well with normal neurology and digestive function.

## 3. Discussion

This case highlights the importance of recognising the “seatbelt sign” and the need to rule out associated injuries. Truncal vascular injuries following blunt abdominal trauma are rare in children with few published reports in literature [4]. Abdominal vascular injuries are less common following blunt trauma with penetrating trauma accounting for 70% to 90% of these injuries. SMV injuries are rare and lethal injuries in children. Case reports or series reporting exclusively on SMV injuries in the paediatric population are lacking in published English literature. SMV injuries following blunt abdominal trauma in children are usually reported in a case mix of other abdominal vascular injuries that include adult patients [4–8]. The recommended management of SMV injuries varies from ligation in haemodynamically unstable trauma patients to primary repair in those with stable physiology [5, 8].

Injuries associated with SBS in children may differ from those commonly reported in adults and are often difficult to diagnose [3, 9]. Delay in diagnosis can result in higher morbidity or mortality. A high index of suspicion is necessary for rapid recognition and intervention which is the key to management of these potentially fatal injuries. Although seatbelts have substantially reduced fatalities associated with motor vehicle accidents, correct use of seatbelts needs to be stressed. When properly worn, with loading point on the anterior superior iliac spine, the same force would not cause internal organ injury as the energy is distributed through the bone. With higher forces, pelvic fractures and lumbosacral spine injuries can occur, while avoiding internal organ injury. Incorrect use of seatbelts is linked to higher chances of injuries in road traffic accidents. Education and greater public awareness about correct use of seatbelts in children is necessary.
